# Enhancing radiosensitivity of boron neutron capture therapy for liver cancer with homologous recombination repair inhibitor

**DOI:** 10.1007/s11604-025-01852-z

**Published:** 2025-08-18

**Authors:** Zih-Yin Lai, Yu-Hsuan Huang, Ting-Yu Zhou, Chi-Ying Lee, Yu-Ming Shiao, Yi-Wei Chen, Fong-In Chou, Jen-Kun Chen, Yung-Jen Chuang

**Affiliations:** 1 School of Medicine, National Tsing Hua University, Hsinchu, Taiwan R.O.C.; 2 Institute of Bioinformatics and Structural Biology, National Tsing Hua University, Hsinchu, Taiwan R.O.C.; 3 Nuclear Science and Technology Development Center, National Tsing Hua University, Hsinchu, Taiwan R.O.C.; 4 GeneReach Biotechnology, Taichung, Taiwan R.O.C.; 5 Department of Heavy Particles and Radiation Oncology, Taipei Veterans General Hospital, Taipei, Taiwan R.O.C.; 6 Institute of Biomedical Engineering and Nanomedicine, National Health Research Institutes, Miaoli, Taiwan R.O.C.

**Keywords:** Hepatocellular Carcinoma, BNCT, RAD51, Autophagy, DNA Repair

## Abstract

**Background:**

Hepatocellular carcinoma (HCC), particularly in recurrent or treatment-refractory cases, often exhibits poor responsiveness to radiation therapy, increasing the risk of radiation-induced liver disease, necessitating innovative treatment approaches. Boric acid-mediated boron neutron capture therapy (BA-BNCT) has emerged as a promising approach for liver cancer. This study aims to improve BA-BNCT efficacy for radioresistant HCC by exploring sensitization agents, enhancing treatment while minimizing irradiation doses and side effects.

**Methods:**

We targeted the DNA homologous recombination repair (HRR) protein RAD51. Before neutron irradiation, a RAD51 inhibitor, B02, was administered to evaluate its sensitization effect on both HepG2 and the radioresistant HepG2R cells. We examined the cell death mechanism, focusing on the expression profile of LC3B after BA-BNCT, to investigate its impact on DNA repair responses, especially on autophagy and apoptosis.

**Results:**

We observed that inhibition of RAD51 led to increased γH2AX, the DNA double-strand break marker. Additionally, combining the RAD51 inhibitor B02 with BA-BNCT resulted in tumor cell arrest in the G_0_/G_1_ phase, indicating altered cell cycle regulation. In exploring cell death mechanisms, we observed increased autophagy following BNCT, potentially as a response to cellular stress induced by DNA damage in tumor cells. The combination of B02 and BA-BNCT significantly disrupted autophagic flux and promoted apoptosis in the tumor cells.

**Conclusions:**

Combining a RAD51 inhibitor with BA-BNCT significantly enhances the anti-tumor efficacy against radioresistant HCC and parental HCC cells. This proof-of-concept study suggests that the combination treatment can achieve comparable or superior therapeutic effects using lower radiation doses, thereby reinforcing the potential of BNCT for treating recurrent HCC.

**Supplementary Information:**

The online version contains supplementary material available at 10.1007/s11604-025-01852-z.

## Introduction

Liver cancer, particularly hepatocellular carcinoma (HCC), has a global 5-year survival rate of 18% [1]. Treatments such as surgery, ablation, and TAE may not always be feasible or effective [2]. Although HCC is not inherently radioresistant, the high radiosensitivity of the surrounding normal liver tissue limits the delivery of curative radiation doses. Consequently, conventional X-ray radiotherapy is often avoided due to the risk of liver toxicity, particularly in recurrent cases where re-irradiation becomes challenging following treatments such as resection, ablation, or embolization [3–6]. Additionally, the occurrence of radiation-induced liver disease (RILD) restricts the escalation of radiation dose and the possibility of re-irradiation. Thus, it is crucial to strike a balance between maximizing the effectiveness of the treatment and minimizing damage to healthy liver tissue.

Boron Neutron Capture Therapy (BNCT) is a promising treatment that uses the boron-10 drug to target cancer cells selectively [7, 8]. When ^10^B-enriched cells are exposed to neutron irradiation, alpha particles and lithium nuclei are generated to destroy cancer cells while sparing the surrounding healthy tissue [9]. The most commonly used boron-containing drug in clinical practice is boronophenylalanine (BPA) [10, 11]. However, BPA is not considered a suitable boron drug for HCC in BNCT. Although BPA is the most widely used boron carrier in clinical BNCT, its application in liver cancer remains limited. While L-type amino acid transporter 1 (LAT1) expression in HCC may facilitate BPA uptake, preclinical studies have shown suboptimal tumor selectivity in liver cancer models [12, 13]. In contrast, boric acid (BA) has demonstrated a more favorable tumor-to-liver boron distribution profile in animal studies, supporting its use in experimental BNCT for HCC [14, 15]. Given these reasons, BA-mediated BNCT (BA-BNCT) currently represents a more practical boron delivery strategy for liver cancer.

Previous studies have shown that BNCT causes extensive DNA damage, especially double-strand breaks (DSBs). The damaged DNA in treatment-resistant cancer cells may be repaired primarily through the homologous recombination (HR) pathway [16]. Under optimal treatment conditions, the severity of DNA damage shall overwhelm repair mechanisms in the resistant cancer cells, accumulating irreparable damage and ultimately inducing cancer cell death [17]. Thus, the effectiveness of BNCT relies on its ability to cause critical levels of DNA damage that surpass the cancer cell's repair capacity.

DNA damage activates autophagy, a self-regulatory process that eliminates damaged components to restore cellular homeostasis and preserve genomic stability [18, 19]. Essential proteins such as LC3/GABARAP serve as autophagosome markers, while receptors like Sequestosome-1 (SQSTM1, also known as p62) direct ubiquitinated proteins to be degraded by lysosomal enzymes [20, 21]. Autophagy deficiency leads to genomic instability, impaired clearance of damaged organelles, reactive oxygen species accumulation, and defective DNA repair [22, 23]. Studying DNA damage-induced autophagy could offer insights into the unique action mechanism of BNCT and help address associated pathologies.

Building on our previous work that demonstrated the potential of BA-BNCT for treating radioresistant HCC [16], the present study introduces a novel radiosensitization strategy by combining BA-BNCT with RAD51 inhibition using B02. This approach aims to enhance therapeutic efficacy through dual modulation of DNA damage repair and autophagic signaling. In contrast to our earlier findings, this study confirms the sensitizing effect of B02 and reveals mechanistic differences between HepG2 and radioresistant HepG2R cells, particularly in DNA damage repair responses and autophagic flux disruption. These insights offer a new rationale for integrating DNA repair inhibitors with BNCT and may guide precision strategies for treating refractory liver cancer.

## Materials and methods

### Cells and cell culture

The human hepatocellular carcinoma HepG2 cell line, authenticated as the C3A/HepG2 (ATCC No. CRL-10741), and its derivative radiation-resistant HepG2R cell line were established in our previous study and maintained according to the same protocol [16]. Cells were cultured in DMEM supplemented with 10% heat-inactivated fetal bovine serum, 100 unit/mL penicillin, 100 μg/mL streptomycin, and 0.25 μg/mL amphotericin B in a humidified incubator at 37 °C with 5% CO_2_.

### ***Cell viability assay and IC***_***50***_*** determination***

HepG2 and HepG2R cell lines were seeded with 5 × 10^3^ cells per well in 96-well plates. Following a 24-h incubation period, the B02 (RAD51 inhibitor; Selleck Chemicals, Houston, TX, USA) was diluted to the desired concentration and added to the wells. After 72 h of treatment, Cell Counting Kit-8 (CCK-8 kit; Dojindo, Kumamoto, Japan) cell proliferation reagent was added and incubated for 1 h. Absorbance readings were then taken at a wavelength of 450 nm, and the IC_50_ values were subsequently calculated using GraphPad Prism 9 (San Diego, CA, USA).

### Boron solution and boron uptake concentration

^10^B-enriched boric acid (BA; 99% ^10^B) was purchased from Aldrich Inc. (Darmstadt, Germany), prepared as a stock solution at a 6000 μg ^10^B/mL concentration, sterilized using a 0.22 μm filter, and stored at 4 °C. As described in our previous study [16], the ^10^B concentration was 58.45 ± 2.08 ppm for HepG2 cells and 59.13 ± 4.82 ppm for HepG2R cells after a 30-min incubation with 25 μg ^10^B/mL BA. This 30-min incubation period was selected because boron accumulation in both HepG2 and HepG2R cells reached a near-saturation level within this timeframe.

### Drug treatment and neutron irradiation

Before neutron irradiation, 4 × 10^5^ cells were seeded in a 6-well plate. Twenty-four hours after seeding, the B02 and combination groups received an additional 10 μM of B02 and were cultured for 24 h. Cells were exposed to 25 μg ^10^B/mL BA for 30 min before neutron irradiation based on our previous study [16]. Subsequently, the culture medium was aspirated, and the cells were transferred to the Tsing Hua Open-pool Reactor (THOR; National Tsing Hua University, Hsinchu, Taiwan). The BNCT procedure followed the previous experiment [16]. The cells were irradiated with 1 Gy, and the detailed physical dose rates of THOR are provided in Supplementary Table 1.

### Colony formation assay

Following irradiation, cells were detached using trypsin and seeded at a density of 3 × 10^3^ cells per well in a 6-well plate. Subsequently, the cells were cultured for 10–14 days until individual colonies reached a size of more than 50 cells. Following cell growth, the colonies were rinsed with PBS and then fixed with 95% methanol (Merck, Darmstadt, Germany) for 5 min. Subsequently, they were stained with 0.1% crystal violet (Sigma Aldrich, Steinheim, Germany) for 30 min.

### Western blot assay

At 4-, 10-, and 24-h post-irradiation, both HepG2 and HepG2R cells were harvested according to the detailed protocol of previous research [16]. Cells were lysed using RIPA buffer (Roche, Indianapolis, IN, USA), and the total protein was subjected to 10% SDS–PAGE, followed by transfer onto PVDF membranes (Millipore, Darmstadt, Germany). Membranes were blocked with 3% BSA and incubated overnight at 4 °C with primary antibodies. After washing, membranes were incubated with horseradish peroxidase-conjugated secondary antibodies (GE Healthcare, Buckinghamshire, UK; GeneTex, Irvine, CA, USA) for 2 h. Protein bands were visualized using the ImageQuant LAS 4000 mini-imaging system (GE Healthcare, Chicago, IL, USA). The KU80 (#2180), KU70 (#2180), p62 (#5114) and RAD51 (#2662) primary antibodies were purchased from Cell Signaling Technology (Danvers, MA, USA). GAPDH (GTX627408) and LBC3B (GTX127375) primary antibodies were purchased from GeneTex (Hsinchu, Taiwan).

### Immunocytochemistry (ICC)

The experimental process followed a previous study with modifications [24]. Immediately after neutron irradiation, 6 × 10^3^ HepG2 and HepG2R cells were separately reseeded on sterile glass coverslips. At 10 and 24 h after irradiation, cells were harvested. γH2AX (Ser139; #9718, Cell Signaling Technology, Danvers, MA, USA) antibody was incubated overnight at 4 °C. Then, a secondary antibody, anti-rabbit IgG-DyLight 488 (Jackson, West Grove, PA, USA), was added and incubated at 37 °C for 2 h. Nuclei were stained using Hoechst 33342 (Invitrogen, Carlsbad, CA, USA) for 15 min. Immunofluorescence images were captured using an LSM800 confocal microscope (Zeiss, Oberkochen, Germany).

### Cell cycle assay

The experimental process followed a previous study with modifications [24, 25]. HepG2 and HepG2R cells were harvested 4 and 10 h after irradiation, then washed twice with PBS. Next, a dropper was used to add 5 mL of −20 °C 80% ethanol to the cells while gently vortexing. Cells were stained using BD Pharmingen™ 7-aminoactinomycin D (7-AAD) staining solution (559925; BD Biosciences) in BD Pharmingen™ Staining Buffer (554656; BD Biosciences) and then analyzed using a CytoFlex flow cytometer system (Beckman, Indianapolis, IN, USA). Data were further analyzed using CytExpert software (Beckman).

### Caspase-3 apoptosis assay

The experimental procedure was carried out per the PE Active Caspase-3 protocol apoptosis kit from BD Biosciences (San Diego, California, USA). Caspase-3 detection was performed on HepG2 and HepG2R cells, which were harvested 24 h after irradiation; the cells were washed with PBS and stained with PE rabbit anti-activator. Then, the stained cells were passed through a 35 μm nylon mesh cell strainer (Falcon, Reynosa, Tamaulipas, Mexico) and analyzed using a flow cytometry system (CytoFlex; Beckman, Indianapolis, IN, USA). CytExpert was used for further data analysis (Beckman).

### Statistical analysis

Experimental data are presented as mean ± standard deviation (SD). All experiments were analyzed by GraphPad Prism 9 (San Diego, CA, USA) using an unpaired Student's *t*-test. The calculated value is considered statistically significant when* p* < 0.05.

## Results

### RAD51 inhibitor B02 exhibits radio-sensitizing properties in BNCT

**Fig. 1:**
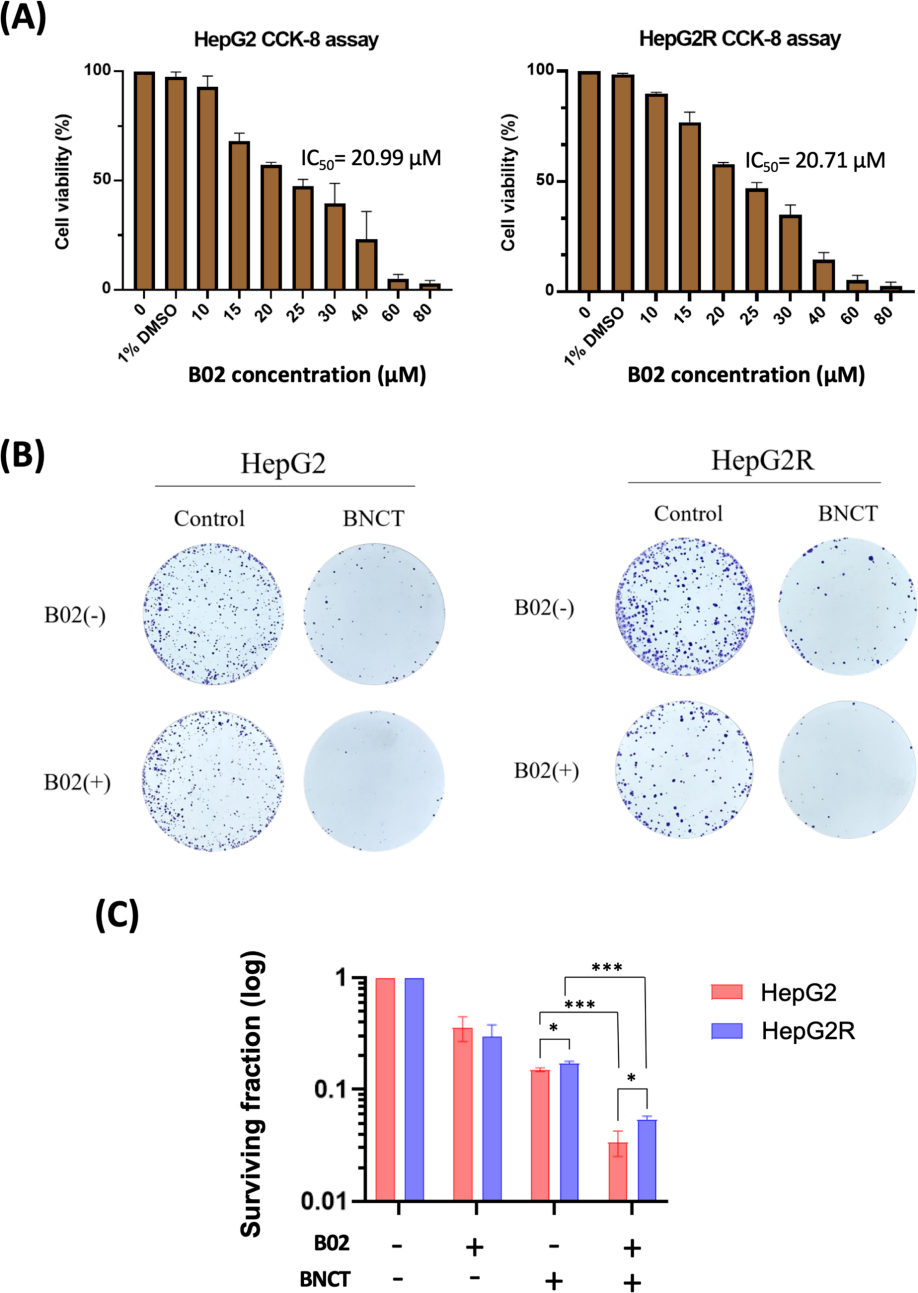
Combined BNCT and B02 treatment effectively eliminates both HepG2 and HepG2R. **A** The resulting IC_50_ values of B02 were 20.99 μM for HepG2 cells and 20.71 μM for HepG2R cells. **B** The colony formation assay assessed cell viability after B02, 1 Gy BNCT, and their combination. **C** Colonies were stained using crystal violet, and the surviving fraction was quantified. (*p < 0.05, ***p < 0.001, mean ± SD)

B02, a RAD51 inhibitor, disrupts the interaction with damaged DNA, reducing RAD51 foci formation and weakening homologous recombination repair (HRR) [26]. It’s also utilized in liver cancer studies [27]. In this study, the IC_50_ of B02 in eliminating HepG2 and HepG2R cells was 20.99 μM and 20.71 μM, respectively (Fig. 1A). There was no significant difference in survival rates between the cell lines. The IC_10_ concentration was approximately 10 μM, significantly inhibiting RAD51 foci formation [28]. Therefore, 10 μM of B02 was used for subsequent experiments.

The colony formation assay assessed the cell surviving fraction following the combined treatment with BA-BNCT and B02 (Fig. 1B). The surviving fraction of HepG2 cells treated with B02 alone was approximately 0.57 ± 0.02 compared to the untreated group. The BA-BNCT monotherapy group had a surviving fraction of 0.14 ± 0.05, and the combined treatment group had a surviving fraction of 0.03 ± 0.01. For the radioresistant HepG2R cells, the surviving fraction after adding B02 was approximately 0.48 ± 0.05. The BA-BNCT monotherapy group had a surviving fraction of 0.17 ± 0.02, and the combined treatment group had a surviving fraction of 0.05 ± 0.02 (Fig. 1C).

The results showed that after 1 Gy BA-BNCT irradiation, the group treated with 10 μM B02 more effectively eliminated HepG2 and HepG2R cells than BNCT monotherapy. This finding prompted further research into its superior sensitizer enhancement ratio (SER) [29]. Using the formula in Supplementary Table 2A, the SER was 1.30 for HepG2 and 1.25 for HepG2R, indicating BA-BNCT with B02 is superior. Furthermore, to assess B02’s radiation-sensitizing effect in BA-BNCT, we calculated the relative effect ratio (RER). An RER above 1 indicates sensitization [30]. The RER for HepG2 cells was 5.53, and for HepG2R cells was 3.81 (Supplementary Table 2B), showing B02's strong sensitizing effect, especially in HepG2 cells.

The combined treatment of BNCT and B02 can effectively enhance the elimination of liver cancer cells. B02 inhibits RAD51, reducing HRR. It significantly lowers cell survival rates post-BA-BNCT irradiation, with superior SER and RER values, particularly in HepG2 cells, demonstrating a robust sensitizing effect.

### B02 exacerbates the DNA damage response following BNCT

**Fig. 2:**
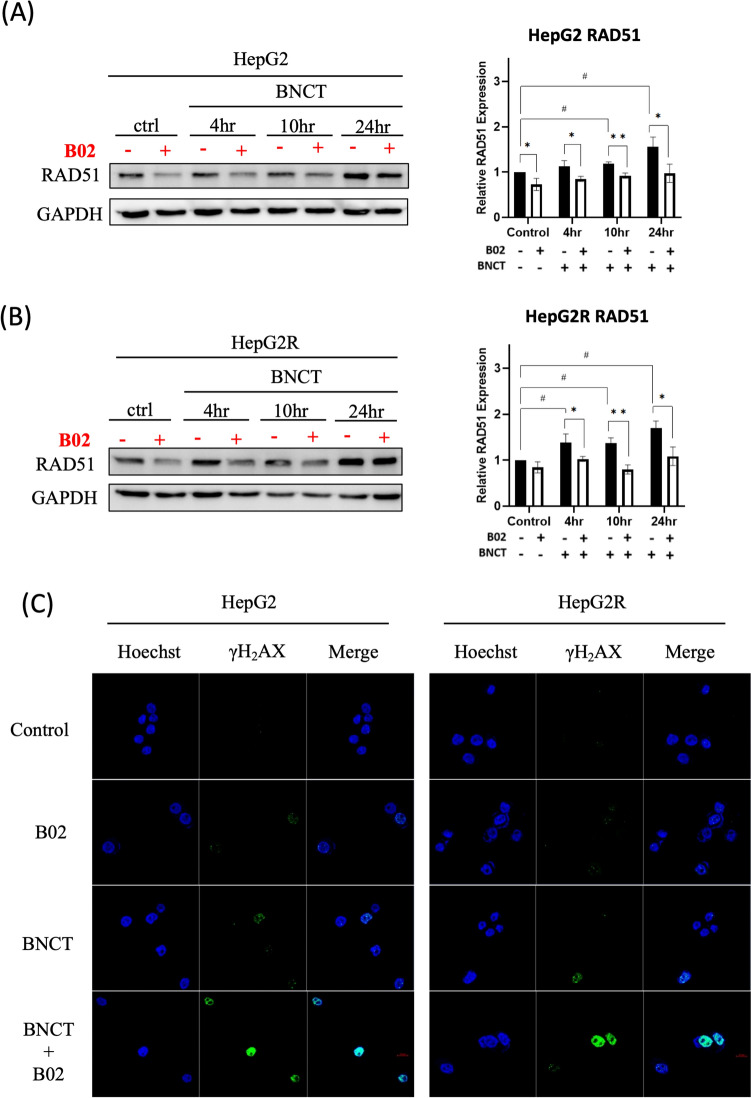
B02 suppresses the HRR pathway and enhances γH2AX expression during BNCT treatment. **A**, **B** The Western blot assay assessed HR-related protein, RAD51, post-BNCT in HepG2 and HepG2R cells, quantified by using ImageJ and normalized by GAPDH. The relative expression of the untreated control was set to 1.00. **C** The representative images depict γH2AX foci (DNA double-strand breaks marker) through immunocytochemistry (ICC). The green fluorescence corresponds to γH2AX, while the blue fluorescence corresponds to nuclei. (*p < 0.05, **p < 0.01, #*p* < 0.05, mean ± SD)

The HRR pathway is crucial in DNA damage repair in cancer radiation biology. Western blot results showed the relative expression level of RAD51 in HepG2 cells was significantly increased by 0.18 ± 0.02- and 0.55 ± 0.12-fold compared to untreated control at 10 and 24 h after BNCT irradiation, respectively (Fig. 2A). Interestingly, HepG2R cells exhibited earlier RAD51 upregulation at 4 h, which was significantly increased by 0.38 ± 0.10-, 0.37 ± 0.06-, and 0.69 ± 0.08-fold compared to untreated control at 4, 10, and 24 h, respectively, indicating potential radioresistance (Fig. 2B).

In the combination treatment of BNCT and B02 group, RAD51 expression in HepG2 was significantly decreased by 0.27 ± 0.07-, 0.26 ± 0.04-, and 0.58 ± 0.17-fold compared to BNCT treatment alone at 4, 10, and 24 h post-BNCT, respectively (Fig. 2A). On the other hand, the RAD51 expression of HepG2R was significantly decreased by 0.35 ± 0.11-, 0.57 ± 0.08-, and 0.61 ± 0.14-fold, respectively (Fig. 2B). Since inhibition of DNA repair processes exacerbates DNA damage, γH2AX was used as a marker to quantify the extent of DNA double-strand breaks [31]. γH2AX expression was increased in HepG2 and HepG2R cells at 10 and 24 h following BA-BNCT and B02 treatment, with a notable rise at 10 h (Fig. 2C). The combination treatment group had higher γH2AX expression than the monotherapy groups.

In short, B02 sensitizes cells under BA-BNCT, inhibiting the HRR pathway, suggesting a promising therapeutic approach to enhance BA-BNCT effectiveness in hepatic malignancies.

### ***The combined treatment induces G***_***0***_***/G***_***1***_*** arrest in liver cancer cells***

**Fig. 3:**
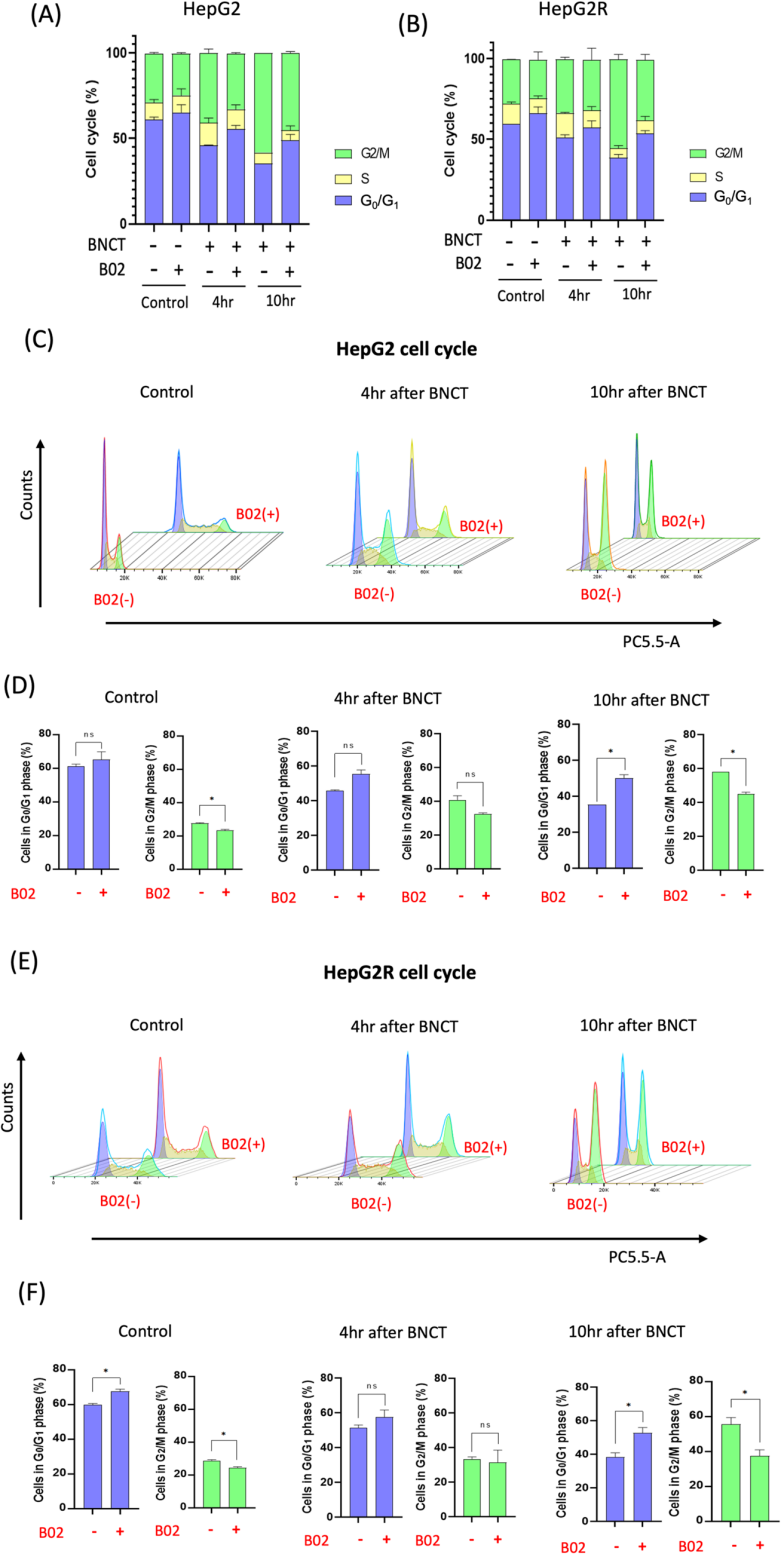
Combined treatment of BNCT and B02 leads to G_0_/G_1_ arrest in HepG2 and HepG2R. **A** Cell cycle distribution was assessed at 4 and 10 h following BNCT treatment, B02 treatment, and their combination in HepG2 cells. **B** Cell cycle distribution was analyzed at 4 and 10 h post-BNCT, B02, and combined treatments in HepG2R cells. **C** The DNA histogram for the cell cycle assay of HepG2 cells was generated using staining with 7-Amino-Actinomycin D (7-ADD). **D** Quantitative analysis of cell cycle assay for G_0_/G_1_ phase and G_2_/M phase in HepG2 cells. **E** The DNA histogram for the cell cycle assay of HepG2R cells **F** Quantitative analysis of cell cycle assay for G_0_/G_1_ phase and G_2_/M phase in HepG2R cells. (*p < 0.05, mean ± SD)

To investigate the subsequent cellular response, we assessed changes in cell cycle distribution after combination treatment. Figures 3A, B showed increased G_2_/M phase population in HepG2 and HepG2R cells at 4 and 10 h after 1 Gy BA-BNCT, indicating DNA repair before mitosis. Since B02 attenuates DNA HRR after BA-BNCT, we examined its effect on cell cycle progression. Figures 3C, E showed HepG2 and HepG2R cell distribution in the G_0_/G_1_, S, and G_2_/M phases. Figures 3D, F provided statistical analysis, focusing on the G_0_/G_1_ and G_2_/M phases at 4- and 10-h post-treatment.

The addition of B02 reduced the G_2_/M phase arrest induced by BA-BNCT, which is evident in both non-irradiated and post-treatment groups. BA-BNCT and B02 combined treatment caused cells to accumulate in the G_0_/G_1_ phase, especially 10 h post-irradiation (Figs. 3C, E). In HepG2 cells, G_2_/M phase cells decreased by 13.79 ± 3.89% while G_0_/G_1_ phase cells increased by 13.05 ± 1.12% (Fig. 3D). In HepG2R cells, G_2_/M phase cells decreased by 18.26 ± 3.65% while G_0_/G_1_ phase cells increased by 14.79 ± 2.16% (Fig. 3F). B02 addition alters cancer cell responses, affecting cell division post-BNCT irradiation and influencing cellular fate.

### B02 inhibits the autophagic flux induced by BA-BNCT

**Fig. 4:**
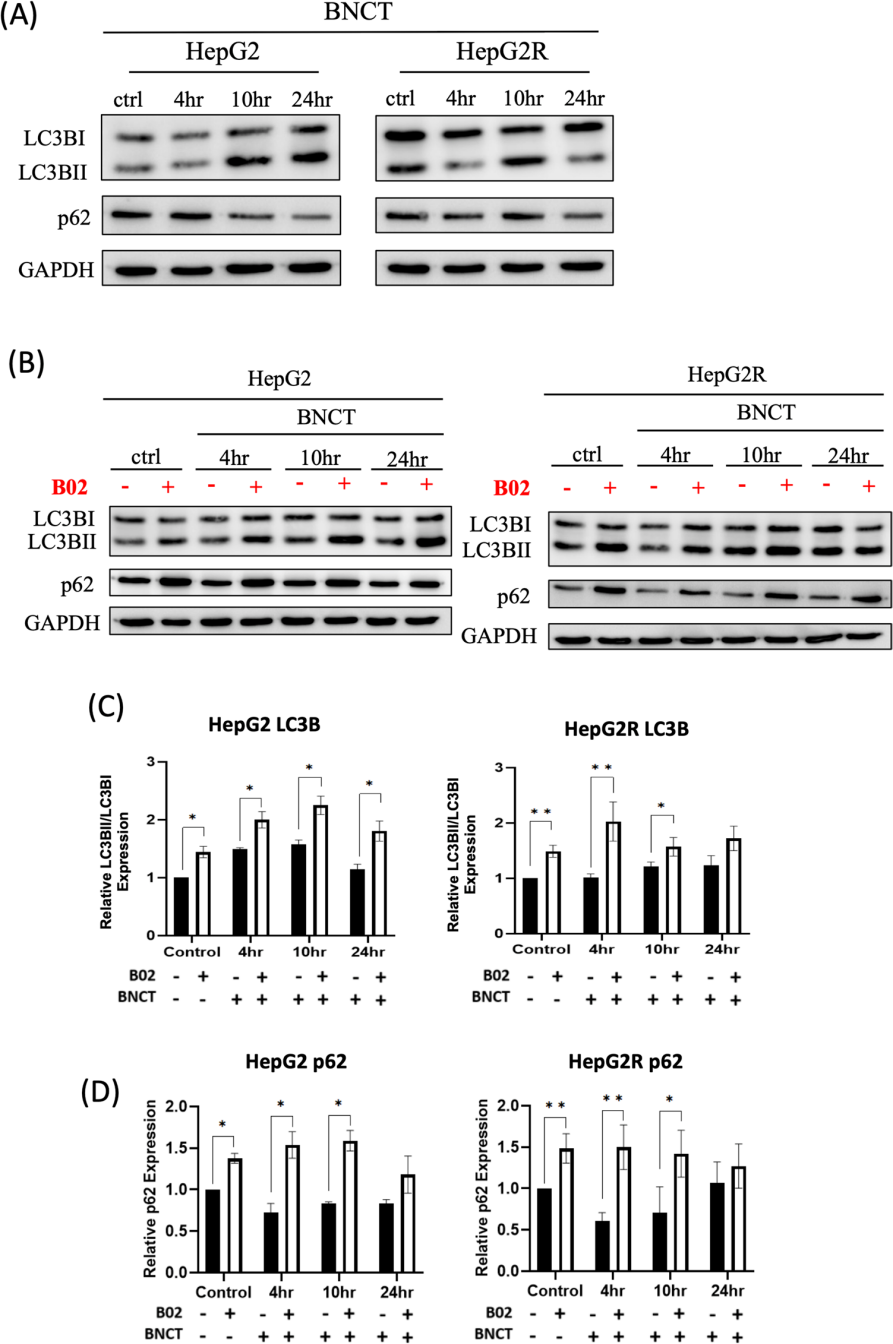
The combined treatment of B02 and BNCT effectively inhibits autophagic flux.** A** The Western blot revealed autophagy-related protein levels after 4, 10, and 24 h of BNCT in HepG2 and HepG2R cells. **B** The Western blot analysis revealed autophagy-related protein levels at 4, 10, and 24 h post-BNCT and combined treatment in HepG2 and HepG2R cells. **C** LC3BII/LC3BI protein ratio was quantified using ImageJ. **D** p62 protein expression was quantified using ImageJ. GAPDH was used as a loading control. The relative expression of the untreated control was set to 1.00. (*p < 0.05, **p < 0.01, mean ± SD)

Studies show autophagy restores cellular homeostasis by degrading damaged DNA [32]. We investigated whether BA-BNCT treatment induces autophagy. LC3B marks early autophagy stages, forming autophagosomes [33]. p62 transports damaged materials to lysosomes for degradation, with decreased p62 indicating increased autophagy [34]. Figure 4A showed a Western blot analysis of LC3B and p62 in HepG2 and HepG2R cells post-BNCT, indicating increased LC3BII/LC3BI ratio and decreased p62, suggesting autophagy induction.

Autophagic flux involves autophagosome formation, lysosome fusion, and degradation, marked by increased LC3BII/LC3BI ratio and decreased p62 levels as substances decompose [35]. To investigate the impact of HRR inhibitor B02 on autophagy induced by BA-BNCT, we assessed the response at 4, 10, and 24 h post-irradiation (Fig. 4B). In the combination treatment group, the LC3BII/LC3BI ratio was increased in HepG2 by 0.50 ± 0.10-, 0.67 ± 0.12-, and 0.65 ± 0.13-fold, and in HepG2R was increased by 1.01 ± 0.20-, 0.35 ± 0.10-, and 0.49 ± 0.16-fold compare to BNCT monotherapy, respectively (Fig. 4C). This suggests that B02 may enhance autophagosome formation, potentially facilitating the encapsulation of damaged DNA for degradation.

The quantification of p62 revealed a cumulative increase after adding B02. At 4, 10, and 24 h, p62 levels in HepG2 was increased by 0.81 ± 0.13-, 0.75 ± 0.08-, and 0.34 ± 0.16-fold, and in HepG2R by 0.88 ± 0.16-, 0.71 ± 0.24-, and 0.20 ± 0.20-fold, respectively (Fig. 4D). This suggests the autophagosome may not be fusing with the lysosome for degradation.

### The apoptotic pathway is upregulated by the addition of B02

The inhibition of autophagic flux prevents the efficient degradation of autophagosomes containing damaged DNA, increasing cellular stress [36]. We speculate that BA-BNCT and B02 treatment causes autophagosome buildup, elevating stress and triggering apoptosis. We assessed Caspase-3 levels to quantify apoptosis post-treatment.

**Fig. 5:**
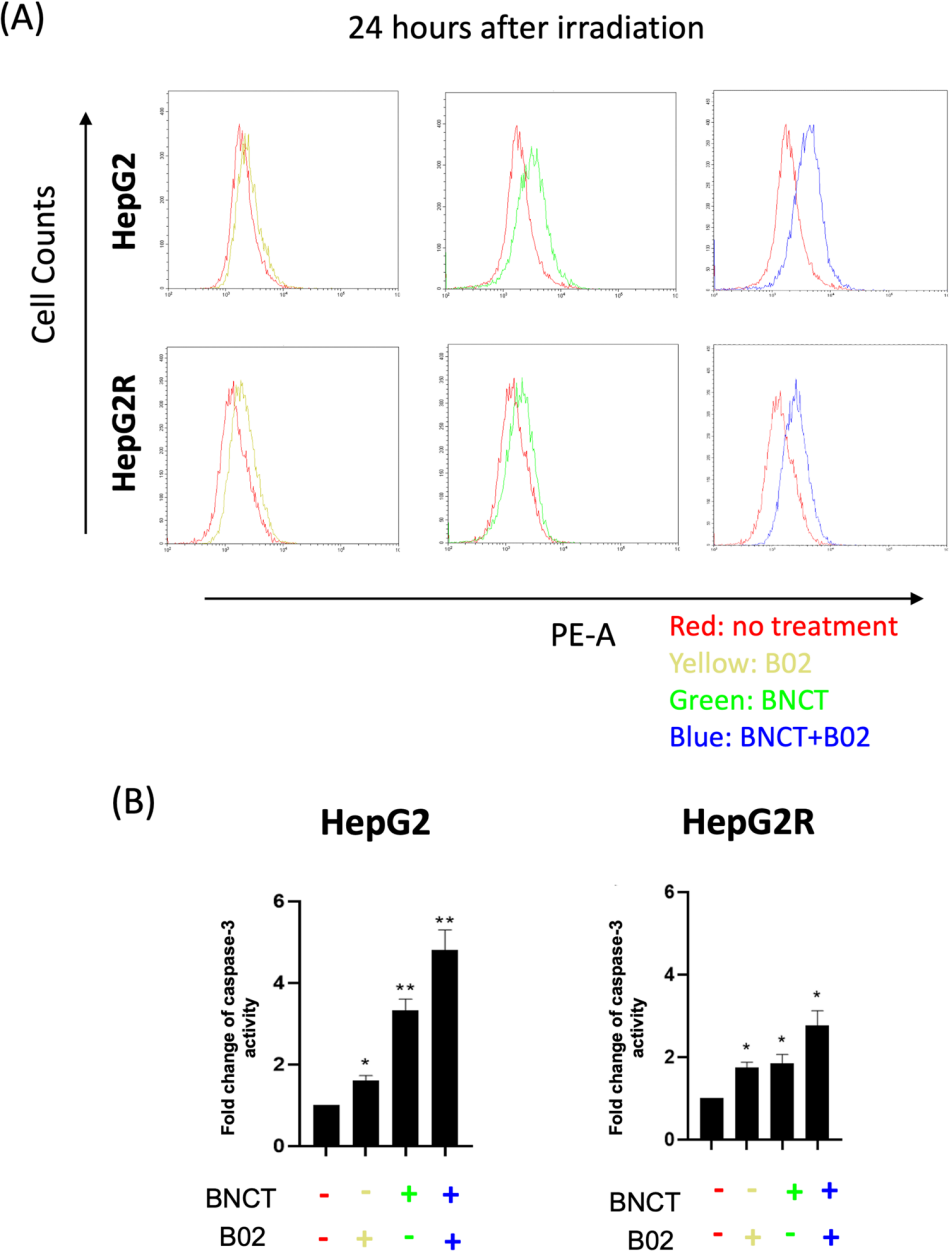
The B02 induces more intensive apoptosis caused by BNCT treatment. **A** The histogram of caspase-3 activity for the apoptosis assay was obtained through PE staining. **B** Quantitative assessment of caspase-3 relative fluorescence intensity. (*p < 0.05, **p < 0.01, mean ± SD)

We monitored Caspase-3 levels at 24 h after BA-BNCT irradiation. Compared to the untreated group (Fig. 5A, red line), the B02-treated group without BA-BNCT (Fig. 5A, yellow line) showed a slight increase in Caspase-3. In HepG2 and HepG2R, Caspase-3 increased by 0.61 ± 0.08- and 0.74 ± 0.09-fold, respectively (Fig. 5B). BA-BNCT monotherapy (Fig. 5A, green line) caused a noticeable increase: 2.32 ± 0.20-fold in HepG2 and 0.85 ± 0.14-fold in HepG2R (Fig. 5B).

After 24 h of BA-BNCT combined with B02 treatment (Fig. 5A, blue line), a substantial increase in Caspase-3 can be observed. The data indicated that in the combined treatment group, HepG2 and HepG2R increased by 3.8 ± 0.34- and 1.7 ± 0.25-fold compared to the untreated group, respectively (Fig. 5B). Both HepG2 and the radioresistant HepG2R exhibited enhanced apoptosis when treated with B02 and BA-BNCT.

## Discussion

This study investigated the integration of the RAD51 inhibitor B02 with neutron capture therapy for liver cancer, emphasizing its sensitizing effect when combined with BA-BNCT. The results demonstrated that this therapeutic agent has substantial potential for enhancing the effectiveness of existing treatment. Key findings revealed that B02 influences DNA damage response pathways, leading to cell cycle changes, autophagy inhibition, and activation of apoptotic pathways.

Animal studies in rat and rabbit liver cancer models have shown that BA-BNCT does not cause significant histological or functional damage to normal liver tissue under therapeutic conditions [14, 15]. The targeted DNA damage response (DDR) constitutes a critical cellular mechanism activated in response to BNCT radiation. This response either initiates DNA repair processes or induces programmed cell death to maintain cellular homeostasis [37]. Our research has shown that inhibiting RAD51, a key protein in the DDR pathway, can enhance the effectiveness of BNCT. Specifically, targeting DDR pathways, such as through RAD51 inhibition, can enhance the cytotoxic effects of radiation in HRR-proficient HCC cells, enabling therapeutic efficacy at lower radiation doses. Although B02 is not tumor-specific in its uptake, previous studies have shown that non-malignant cells are less reliant on HR and thus exhibit reduced sensitivity to RAD51 inhibition under sublethal DNA damage conditions [28, 38]. This differential sensitivity thus presents a potential therapeutic window; however, further investigations are warranted to determine whether off-target effects occur in normal hepatic tissue.

This study also examined the radioresistant HepG2R cell line established in our previous report [16], revealing that under 1 Gy BA-BNCT monotherapy, HepG2R cells exhibited a higher survival rate than HepG2 cells. Notably, the radioresistance in HepG2R was induced by γ-ray exposure, a radiation source distinct from BA-BNCT, suggesting a possible overlap in the cell death mechanisms triggered by these distinct modalities. Regarding the tumor dose effect, previous findings have indicated that both HepG2 and HepG2R cells upregulated the DNA HRR protein RAD51 at 24 h following 2 Gy BA-BNCT irradiation [16]. In the current study, using a lower irradiation dose of 1 Gy, we observed an accelerated RAD51 response: HepG2 cells showed a significant increase at 10 h, whereas HepG2R cells responded even earlier at 4 h post-irradiation. This earlier RAD51 upregulation in HepG2R suggests a more robust HRR capacity, which likely contributes to their enhanced resistance to radiation. These findings highlight the distinct biological responses among tumor subtypes and underscore the need to tailor BNCT strategies according to individual tumor characteristics to optimize therapeutic efficacy. While high-dose BNCT is standard in clinical settings, this study investigated the sensitizing effect of B02 under reduced neutron fluence. This approach may be relevant for treating peripheral tumor areas or microscopic invasions, where neutron delivery is suboptimal. Our findings suggest that B02 could enhance the efficacy of BNCT in these low-dose regions.

Radiotherapy resistance presents a significant challenge to cancer treatment. Genetic testing, particularly through Next-Generation Sequencing (NGS), has become a valuable companion diagnostic tool for predicting patient responses to specific cancer therapies. In our study, we employed this strategy by conducting genome sequencing of HepG2 and HepG2R cells to identify genetic differences and analyze their impact on survival using cBioPortal. From this analysis, we selected four genes—TEDDM1, ZNF648, NAV1, and TASOR2—for further investigation (Supplementary Fig. 1A).

According to the Human Protein Atlas (HPA) database, TASOR2 protein expression is elevated in liver cancer (Supplementary Fig. 1B) and is associated with poorer patient prognosis (Supplementary Fig. 1C). TASOR2 functions as a component of the HuSH complex, which silences transposable elements such as LINE-1 [39]. Disruption of this complex can lead to LINE-1 reactivation, causing DNA damage and potentially promoting tumorigenesis (Supplementary Fig. 1D) [40]. Studies have shown that elevated LINE-1 expression correlates with poor outcomes in liver cancer patients [41], and increased LINE-1 activity is linked to elevated expression of HRR proteins, contributing to treatment resistance [42]. These findings provide critical context for our study, which shows that high expression of TASOR2 and HRR-related proteins, including BRCA1/2 and RAD51, is associated with poor survival in liver cancer (Supplementary Fig. 2A). TASOR2 mutations may activate LINE-1, thereby increasing BRCA1/2 and RAD51 expression and promoting resistance to therapy (Supplementary Fig. 2B). Our results also revealed that DNA repair proteins in radioresistant HepG2R cells are upregulated earlier than in parental HepG2 cells, consistent with NGS data showing that radioresistance is associated with enhanced DNA damage repair at both the genetic and protein levels. These insights highlight the potential of targeting the HRR pathway or LINE-1 activity in refractory liver cancer. In addition to the radiosensitizing effects of HRR-targeted drugs demonstrated in this study, LINE-1 inhibitors such as capsaicin may offer a novel therapeutic approach for hard-to-treat liver cancers [43, 44].

**Fig. 6:**
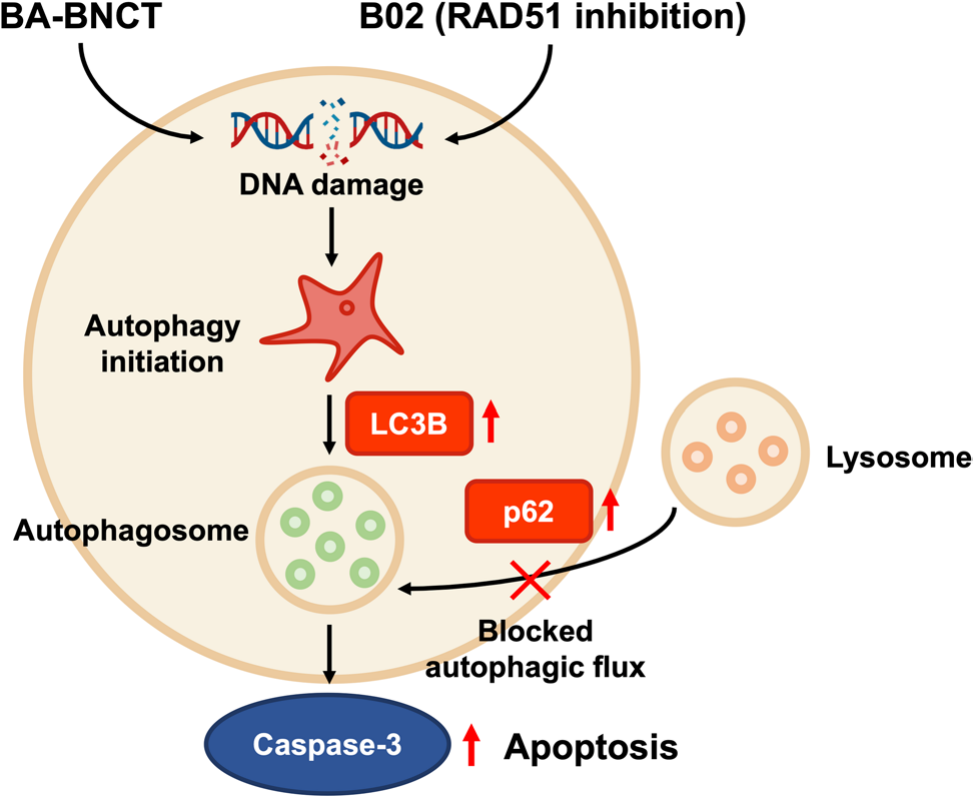
The proposed mechanism of RAD51 inhibition disrupts BA-BNCT-induced autophagic flux and promotes apoptosis

Our findings also indicated that B02 increased the LC3BII/LC3BI ratio, possibly enhancing autophagosome formation. However, the elevated p62 levels might suggest a potential blockage in autophagic flux, possibly disrupting degradation and inhibiting BA-BNCT-induced autophagy in HepG2 and HepG2R cells. The resulting inability to resolve intracellular stress leads to a shift in the cellular response, promoting the activation of apoptotic pathways and culminating in programmed cell death. This concept has been highlighted in the schematic diagram (Fig. 6).

This study thoroughly investigates the therapeutic possibilities of DNA repair inhibition and its integration with BA-BNCT. Utilizing sensitization agents to boost the effectiveness of BNCT shows great promise. This is especially important as it indicates a potential approach to reducing the necessary neutron dose. Although dose reduction presents a theoretical advantage, its clinically relevant application lies in enhancing BNCT efficacy in deep-seated or microinvasive regions of HCC where neutron fluence is suboptimal. Our implementation of reduced-dose conditions in vitro was intended to model these low-fluence zones, thereby providing a framework to evaluate whether B02 can extend BNCT’s therapeutic reach beyond conventional neutron penetration limits. While combining BNCT with sensitizing agents enhanced cytotoxicity in HCC cells, its impact on normal liver tissue remains uncertain, particularly given the non-selective distribution of boric acid and the systemic action of RAD51 inhibitors. Further studies are needed to evaluate potential off-target effects in normal hepatocytes. In addition, the accumulation profile of sensitizers, such as B02, in normal tissues remains poorly characterized, raising concerns about potential adverse events. Furthermore, because low-dose neutron scattering beyond the targeted tumor site is inevitable in BNCT, the concurrent presence of bioactive sensitizers may pose a risk of unintended cytotoxicity in adjacent normal tissues.

While the results are encouraging, several limitations must be acknowledged. First, the study was conducted in vitro, and while the cell line models provide valuable insights, they cannot fully replicate the complex tumor microenvironment found in vivo. Future studies should include animal models to validate these findings and assess the long-term safety and efficacy in a more biologically relevant context. Additionally, identifying optimal sensitization agents requires further exploration, particularly concerning their pharmacokinetics and potential side effects in clinical settings.

In conclusion, this study expands upon previous work by integrating a DNA repair-targeted sensitization strategy into BA-BNCT for HCC. These findings also establish the foundation for additional research on the clinical implementation of this strategy, which could expedite the creation of innovative treatment approaches and contribute to advancements in managing advanced liver cancer.

## Supplementary Information

Below is the link to the electronic supplementary material.

Supplementary file1 (DOCX 811 KB)

## Abbreviations

7-AAD: 7-Amino-Actinomycin D
BA: Boric acid
BNCT: Boron neutron capture therapy
BPA: Boronophenylalanine
BRCA1: Breast cancer 1
BRCA2: Breast cancer 2
DDR: DNA damage response
DSB: DNA double-strand break
DSBR: Double-strand break repair
EBRT: External Beam Radiation Therapy
GAPDH: Glyceraldehyde 3-phosphate dehydrogenase
Gy: Gray
HCC: Hepatocellular carcinoma
HPA: Human Protein Atlas
HR: Homologous recombination
HRR: Homologous recombination repair
IC_50_: 50% Growth-inhibitory concentration
LAT1: L-type amino acid transporter 1
NGS: Next-generation sequencing
γH2AX: Phosphorylated-H2AX
RER: Radiation enhancement ratio
RILD: Radiation-induced liver disease
SER: Sensitizer enhancement ratio
THOR: Tsing Hua Open-pool Reactor
