# Supplementary material for: Enhancing radiosensitivity of boron neutron capture therapy for liver cancer with homologous recombination repair inhibitor
Source: Jpn J Radiol. 2025 Aug 18;44(1):196–208. doi: 10.1007/s11604-025-01852-z (PMC12769638; doi:10.1007/s11604-025-01852-z)
Supplement: Supplementary file 1 — Supplementary file1 (DOCX 811 KB) [file 11604_2025_1852_MOESM1_ESM.docx]

**Supplementary Table 1. Dose rates of Tsing Hua Open-pool Reactor.**

|  | Dose rate (Gy/minute) | |
| --- | --- | --- |
|  | HepG2 | HepG2R |
| Neutron | 1.2×10^-2^ | 1.2×10^-2^ |
| Gamma | 2.4×10^-2^ | 2.4×10^-2^ |
| ^10^B(n,α)^7^Li | 2.48×10^-3^/ppm ^10^B | 2.48×10^-3^/ppm ^10^B |

**Supplementary Table 2. B02 can serve as a radiosensitizer for BNCT.**


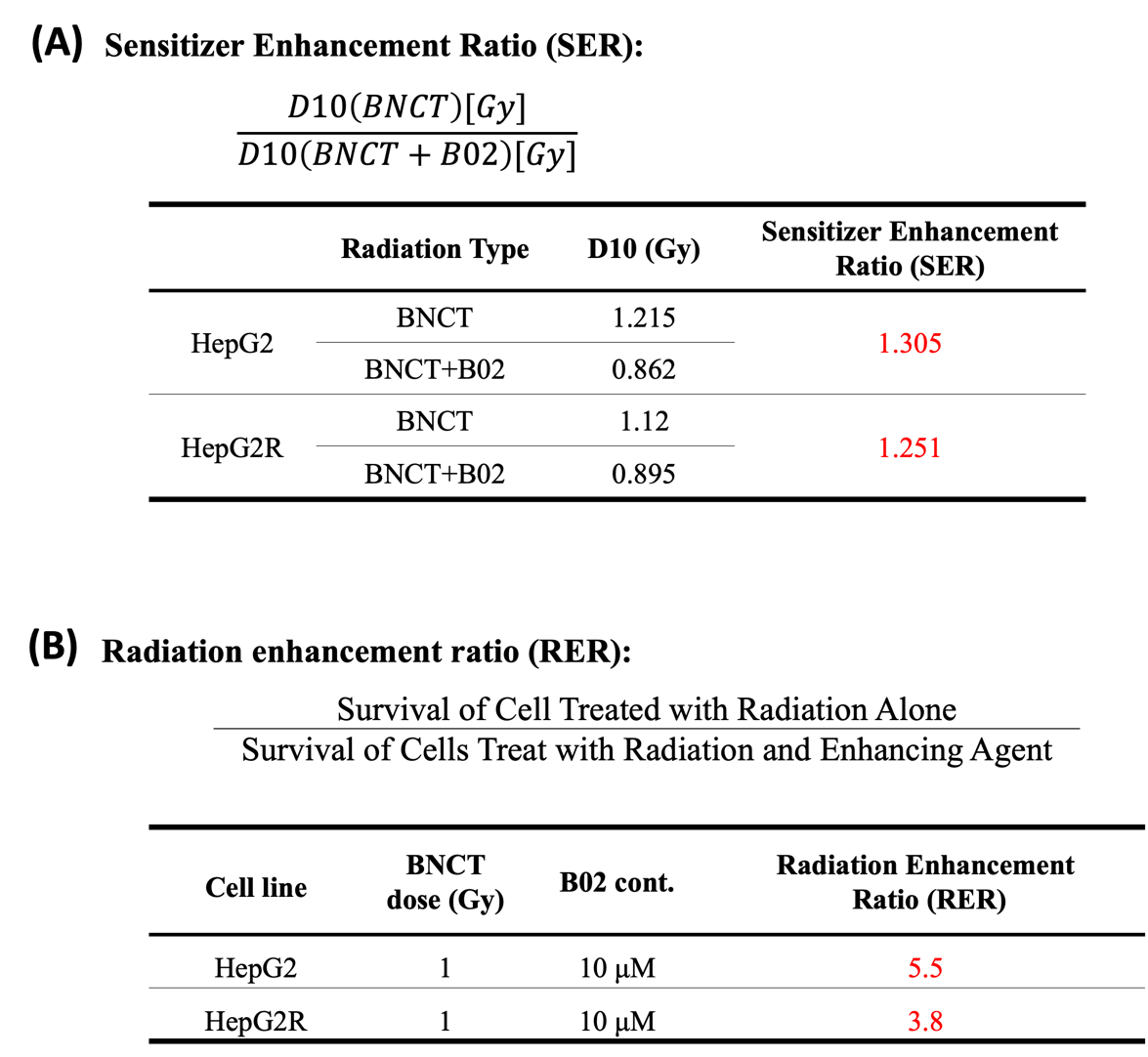


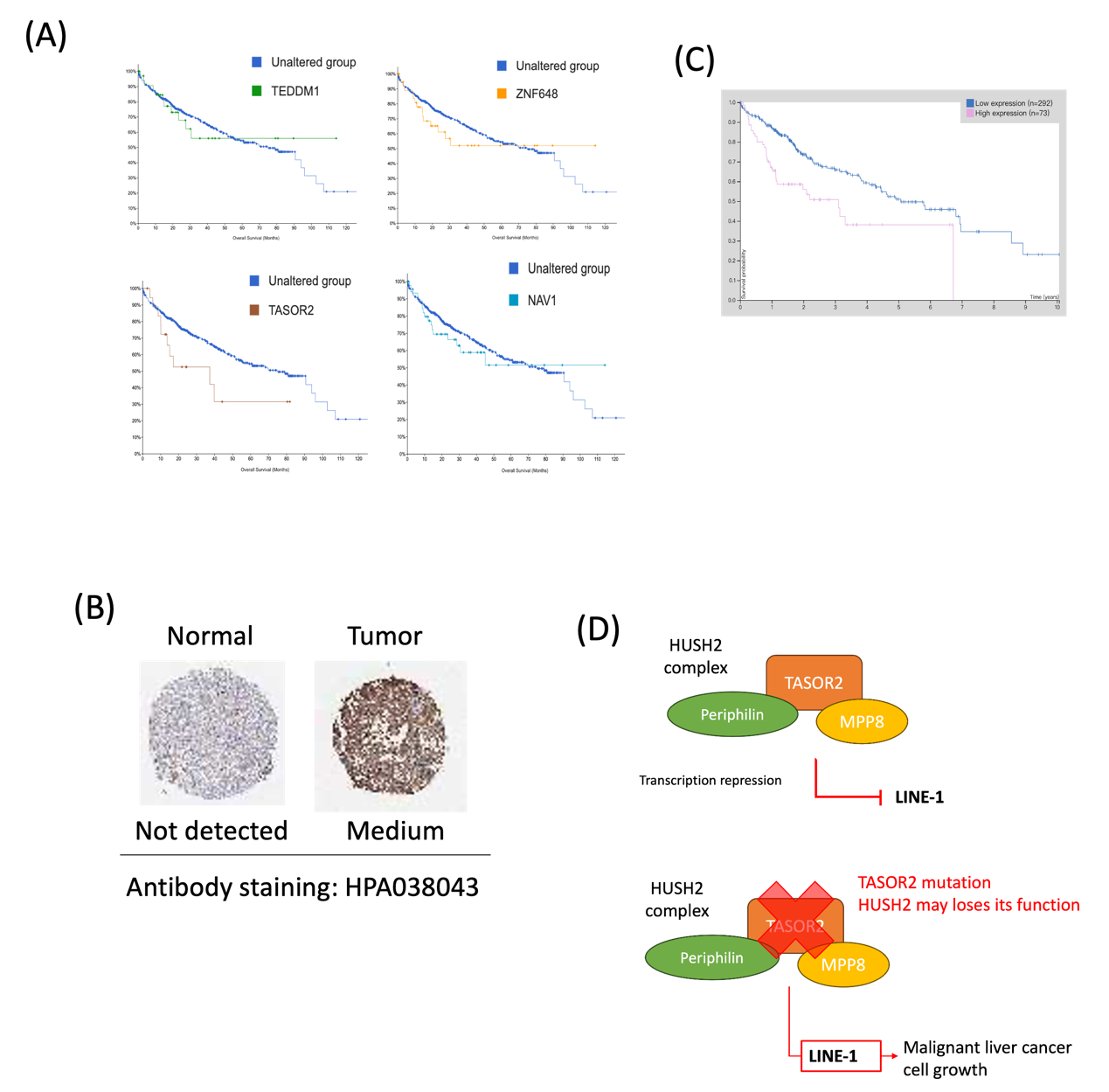


**Supplementary Figure 1.** **Genetic link to radioresistance: TASOR2 mutation is implicated in LINE-1 activation.**

**(A)** Analysis of survival rate of four gene mutations: TEDDM1, ZNF648, TASOR2, and NAV1 in cBioportal. **(B)** The Human Protein Atlas (HPA) database shows that TASOR2 protein is highly expressed in patients with malignant liver cancer. **(C)** Using the HPA database to discover that TASORS protein expression may affect the prognosis of liver cancer. **(D)** Integrated diagram of the molecular mechanism of action of TASOR2. Genomic sequencing of cells was performed using Whole Genome Sequencing (WGS) on the Illumina NextSeq 2000 system (Illumina Inc., USA). Libraries were prepared with the Illumina DNA Prep WGS reagent (Illumina Inc., USA) using the SATLite automation platform (GeneReach Biotech Corp, Taiwan). Major variants in Hep2G and Hep2R cells were analyzed with the DRAGEN Bio-IT Platform (Illumina, Inc., USA), which enables efficient, high-accuracy variant calling and data processing.


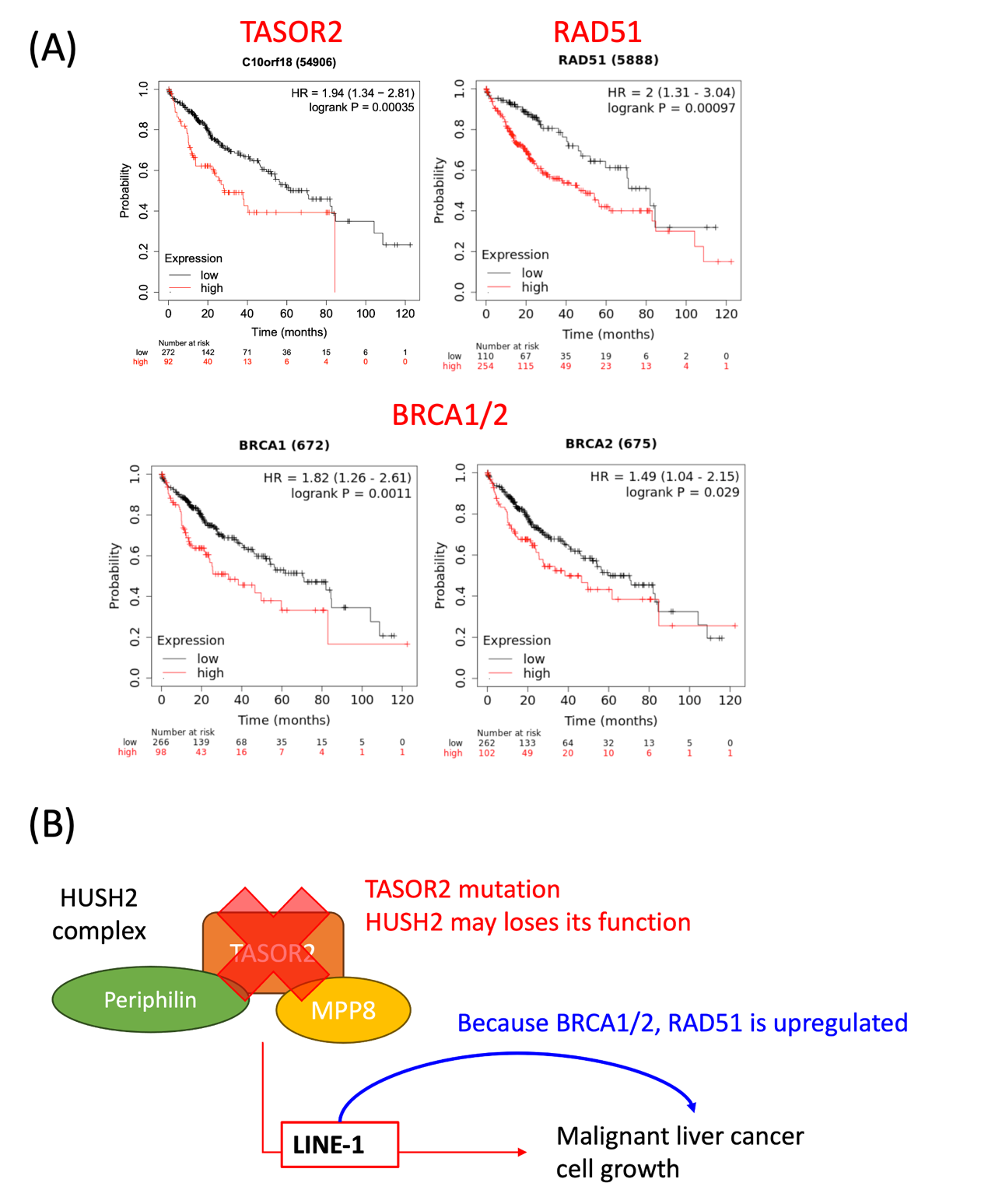


**Supplementary Figure 2. TASOR2 mutations activate LINE-1, increasing the expression of DNA repair proteins like BRCA1/2 and RAD51.**

**(A)** Kaplan-Meier plotter shows that high expression of TASOR2, BRCA1/2, and RAD51 are associated with poor prognosis of liver cancer. **(B)** Integrated chart of TASOR2 mutations leading to activation of downstream homologous recombination (HR) proteins.
